# Investigating *Propionibacterium acnes* antibiotic susceptibility and response to bacteriophage *in vitro* and *in vivo*

**DOI:** 10.3389/fmicb.2024.1424849

**Published:** 2024-06-21

**Authors:** Ruixing Yu, Lingyun Yu, Xiaoli Ning, Yong Cui

**Affiliations:** ^1^Department of Dermatology, China-Japan Friendship Hospital, Beijing, China; ^2^Beijing University of Chemical Technology, Beijing, China; ^3^Peking University China-Japan Friendship School of Clinical Medicine, Beijing, China

**Keywords:** acne vulgaris, *Propionibacterium acnes*, resistance, phage, antibiotics

## Abstract

**Introduction:**

A total of 94 *Propionibacterium acnes* (*P. acnes*) isolates were obtained from a hospital in Beijing to evaluate their susceptibility to erythromycin, clarithromycin, doxycycline, and minocycline. As well as the determination of the effectiveness of *P. acnes* phages *in vitro* and in *P. acnes*-induced lesions mouse model.

**Methods:**

Patients with acne vulgaris (AV) were enrolled from August 2021 to October 2022. Standard methods were employed for specimen collection, culture, and identification of *P. acnes*. Susceptibility testing was conducted using E-strips for erythromycin, clarithromycin, minocycline, and doxycycline. Phage culture and identification followed standard procedures. A mouse model with *P. acnes*-induced skin lesions was established, and data was analyzed using *χ*^2^ test.

**Results:**

The results showed that all isolates were susceptible to minocycline and doxycycline, while 53 (56.4%) and 52 (55.3%) isolates were susceptible to erythromycin and clarithromycin, respectively. Interestingly, younger patients and those with lower acne severity exhibited reduced resistance. Phage cleavage rates ranged from 88.30 to 93.60%. Multilocus sequence typing (MLST) analysis was conducted on eight randomly selected *P. acnes* isolates, and the IA-2 subtype was used in experiments to address *P. acnes*-induced lesions in mice. Phage therapy proved effective in this model.

**Discussion:**

This study highlights the high susceptibility of *P. acnes* to doxycycline and tetracycline, while erythromycin and clarithromycin exhibited elevated resistance. Additionally, *P. acnes* phages demonstrated high cleavage rates and potential effectiveness in treating *P. acnes*-induced lesions. These findings suggest promising avenues for further exploration of phage therapy in acne treatment.

## 1 Introduction

Acne vulgaris (AV) is a prevalent skin disorder characterized by the presence of comedones, papules, pustules, nodules, and cysts. This condition is associated with significant psychological and social consequences, including suicidal ideation, melancholy, anxiety, self-mockery, psychiatric hospitalization, absenteeism, and job-related issues (Samuels et al., 2020). *Propionibacterium acnes* (*P. acnes*) colonization of the follicular infundibulum and sebaceous duct is recognized as a primary contributory factor in AV, triggering inflammatory events by activating innate and adaptive immunity (Jeremy et al., 2003). *P. acnes* is also implicated in abnormal keratinization and differentiation of epidermal keratinocytes, leading to localized inflammation and potential scarring through chemotactic agents and proinflammatory cytokines (Dessinioti and Katsambas, 2010). Treatment of AV often involves anti-inflammatory and antibacterial medications, with antibiotics commonly prescribed to limit or eliminate *P. acnes* colonization and reduce the production of proinflammatory mediators (Thiboutot et al., 2009). Additionally, the treatment of AV also imposes a significant economic burden on the country (GBD 2021 Forecasting Collaborators, 2024).

Commencing in the 1970s, *P. acnes* has exhibited discernible signs of antibiotic resistance (Leyden, 1976). Since the 1980s, a diminishing susceptibility of *P. acnes* strains to various medications prescribed for AV patients has been observed, highlighting a noteworthy concern regarding *P. acnes* antibiotic resistance within the AV population (Xu and Li, 2019). This resistance poses potential risks, including therapy failure, disruption of the natural mucocutaneous microbiota, and the potential onset of local or systemic opportunistic infections. In response to this challenge, emerging alternative therapies, such as bacteriophages, antimicrobial peptides, and naturally generated antibodies, are currently under investigation to counteract *P. acnes* antibiotic resistance.

Bacteriophages, in particular, are considered innovative elements by the US National Institutes of Health (NIH) and are being explored as a potential solution to prevent antibiotic resistance in the context of AV. Distinguishing themselves from antibiotics, phage therapy presents significant advantages, including a natural origin with minimal environmental impact, positive safety profiles, good tolerance, low cost, ease of isolation, widespread distribution at high concentrations, host specificity, and limited cross-resistance (Castillo et al., 2019). Despite these potential benefits, there has been a notable absence of studies examining the capacity of *P. acnes* phages to eradicate *P. acnes in vitro* and *in vivo* tests until now.

The primary aim of this study was to augment the understanding of *P. acnes* isolates obtained in Beijing, China. The susceptibility profile of *P. acnes* in the Beijing region has not been previously explored. To address this gap, the susceptibilities of 94 *P. acnes* isolates to erythromycin, clarithromycin, doxycycline, and minocycline were investigated, utilizing specimens obtained from a hospital in Beijing. Additionally, the efficacy of *P. acnes* phages against *P. acnes* isolates *in vitro* and *P. acnes*-induced lesions in mice was evaluated.

## 2 Materials and methods

**Figure 1:**
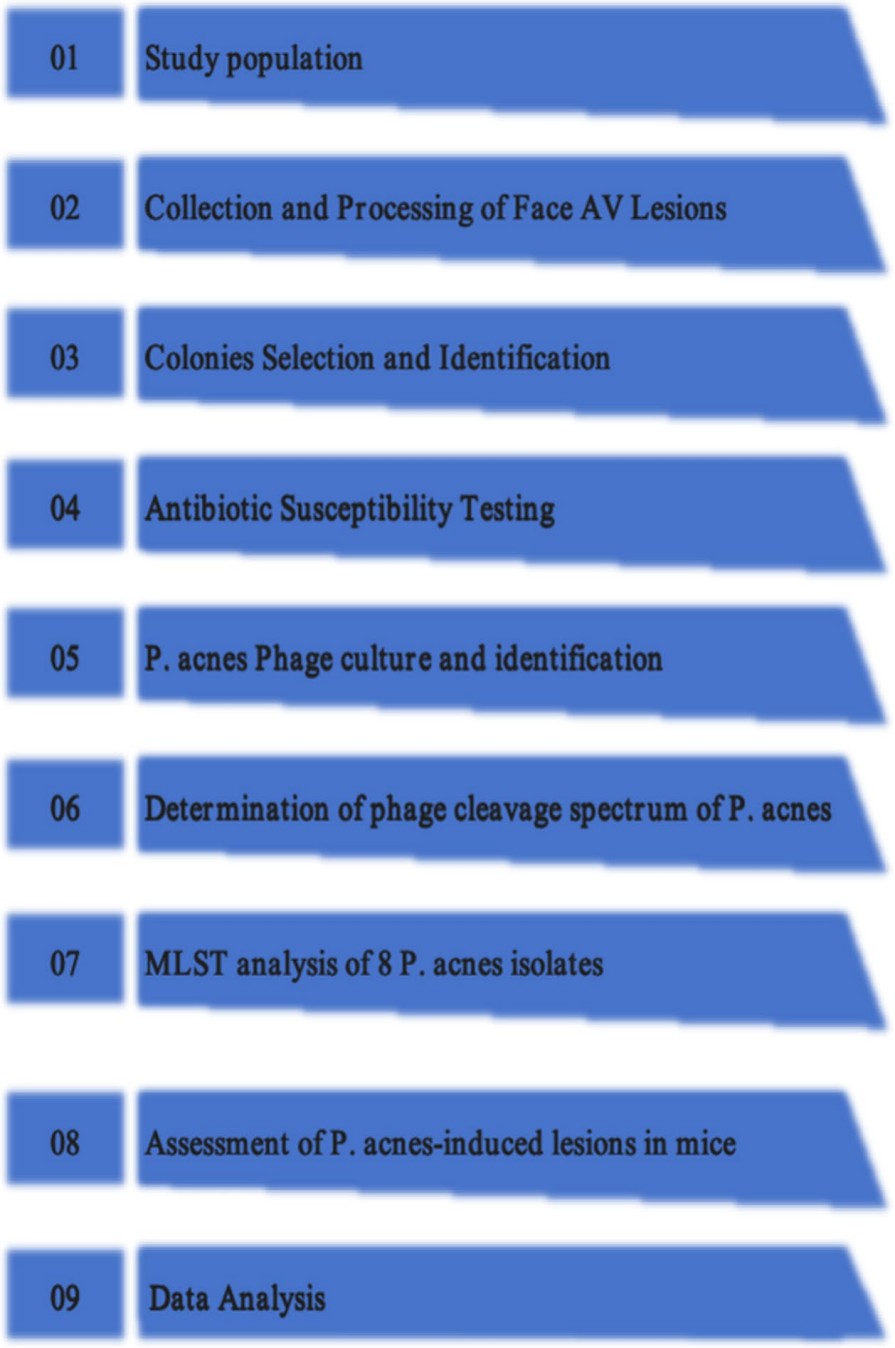
Flow chart to methodology.

The materials and methods were shown in Figure 1.

### 2.1 Study population

This research constituted a prospective and single-center study spanning from August 2021 to October 2022. The dermatology outpatient clinic of a hospital served as the exclusive enrollment site for patients diagnosed with AV during the stipulated period. Patients meeting the following criteria were considered for inclusion: (1) Age between 12 and 50; (2) AV grade 2–4, determined using the established acne grading scheme (Yu et al., 2022). Basic clinical data, encompassing age, gender, AV grades, and collection time, were systematically gathered at the point of patient enrollment or subsequently retrieved from consultation records. Before the initiation of the study, comprehensive informed consent was obtained from all participating patients. For patients below 18 years old, explicit consent was also procured from their parents, ensuring adherence to ethical standards and regulatory protocols.

### 2.2 Collection and processing of face AV lesions

Lesions on the face, including papules, pustules, and cysts associated with AV, underwent a meticulous collection procedure. A needle was employed for puncturing, followed by compression using two swabs. Interstitial fluid was then carefully collected using a swab. This collected fluid was promptly immersed in a transport medium (MT0401, Yocon, China) and transported for subsequent culturing. Within a stringent timeframe of 24 h post-collection, all samples underwent processing. The samples were meticulously placed onto solid medium plates composed of Brain Heart Infusion (BHI) medium from Qingdao Hope Bio-Technology Co. Ltd., Qingdao, China. The incubation was conducted anaerobically for a duration of 7 days at 35°C, facilitated by AnaeroGen^TM^ from Oxoid Ltd., Basingstoke, UK, under anaerobic conditions.

### 2.3 Colonies selection and identification

Typical colonies of *P. acnes* were discerningly chosen based on morphological and Gram-stained properties. These colonies exhibited distinct characteristics, appearing grayish-white and localized to colonies measuring 0.5 mm in diameter. The bacteria displayed pleomorphic Gram-positive bacilli features (Sabatini et al., 2008). For the conclusive identification of bacterial species, a rapid anaerobic bacterial identification system (RAPID ID 32A; bioMe’rieux SA, Marcy l’Etoile, France) was judiciously employed. Pure strains of *P. acnes* were diligently preserved by storing them at −80°C, ensuring their viability and integrity for subsequent analyses.

### 2.4 Antibiotic susceptibility testing

The methodology adhered to the guidelines set forth by both the Clinical and Laboratory Standards Institute (CLSI) and the National Committee for Clinical Laboratory Standards. E-strips for erythromycin, clarithromycin, minocycline, and doxycycline (AB Biodisk, Solna, Sweden) were employed in the susceptibility testing, conducted on a Brain Heart Infusion (BHI) solid medium plate under anaerobic conditions at 37°C (National committee for clinical laboratory standards methods for antimicrobial susceptibility testing of anaerobic bacteria, 2004). The *E*-test provided MICs, defined as the point on the scale at which the ellipse of growth inhibition intercepted the strip. A MIC below the established breakpoint value was indicative of susceptibility to the respective antibiotic. Susceptibility criteria for the four antibiotics were defined as follows (National committee for clinical laboratory standards methods for antimicrobial susceptibility testing of anaerobic bacteria, 2004; Mendoza et al., 2013): Doxycycline and minocycline: ≤4 μg/mL; Erythromycin: ≤0.5 μg/mL; Clarithromycin: ≤0.5 μg/mL.

### 2.5 *Propionibacterium acnes* phage culture and identification

According to the prescribed protocol (Lam et al., 2021), execute the following method was executed: (1) Place 100 μL of each of the 20 *P. acnes* isolates into 10 mL sterile tubes, and add 5 mL of phage buffer (consisting of 10 mM Tris, pH 7.5, 10 mM MgSO_4_, 68.5 mM NaCl, and 1 mM CaCl_2_); (2) Cultivate the samples under anaerobic conditions at 37°C for 72–96 h; (3) Perform a 10 min centrifugation at 11,000 rpm; (4) Filter the resulting supernatants through 0.22 μm filters to remove any residual bacterial cells; (5) Utilize the double-layer agar method to isolate a bacteriophage from the *P. acnes* filtrate, observing plaques on plates after 48 h of anaerobic incubation at 37°C; (6) Introduce *P. acnes*, gather a singular plaque using a sterile pipette tip, and propagate it in a fresh culture; (7) Consecutively purify the phages using the double-layer agar method after proliferation; (8) Store the thoroughly purified phages at −80°C.

### 2.6 Determination of phage cleavage spectrum of *Propionibacterium acnes*

In a meticulously executed procedure, *P. acnes* isolates were uniformly distributed on a solid Brain Heart Infusion (BHI) medium plate and anaerobically cultured at 37°C for 72–96 h. Subsequently, individual colonies were transferred to a 5 mL BHI liquid medium test tube and anaerobically cultured at 37°C for an additional 72–96 h. To initiate the proliferation of phages, 100 μL of a phage proliferation solution was introduced and anaerobically cultured at 37°C for 8–10 h until the culture solution achieved clarity with discernible bacterial fragments. A centrifugation step at 11,000 rpm for 10 min facilitated the collection of the phage proliferation solution from the supernatant. In the subsequent phase, a fresh host bacteria solution (300 μL) was added to 5 mL liquid BHI semi-solid medium at 40–50°C. This mixture was poured onto a solid BHI medium plate, and after solidification, the bottom plate was marked with dividing lines. Finally, 1 μL of freshly filtered phages was strategically dropped at various positions on the plate. Upon natural drying and subsequent incubation in a 37°C environment for 16–18 h, clear spots manifested, unequivocally indicating the phage’s capacity to lyse the *P. acnes* solution. This method adheres to high standards of professionalism and academic rigor, ensuring a meticulously stratified methodology.

### 2.7 Multi-locus sequence typing (MLST) analysis of eight *Propionibacterium acnes* isolates

According to the previously reported (Mak et al., 2013), MLST analysis was conducted on eight randomly selected *P. acnes* isolates out of the 94 isolates in this study.

### 2.8 Assessment of *Propionibacterium acnes*-induced lesions in mice

According to the methods reported in previous literature (Rimon et al., 2023), a mouse model with *P. acnes*-induced skin lesions was established. The IA-2 subtype of *P. acnes* isolate was chosen for the *P. acnes*-induced lesions mice model. This study used SPF-grade ICR mice, all male and 7–8 weeks old. Each experimental group included three mice. In Group A, mice received saline injections; in Group B, mice were injected with *P. acnes* isolates of IA-2 subtype without phage treatment. Group C mice were injected with *P. acnes* isolates of IA-2 subtype with phage treatment. The treatment involved the application of phage 15 to address the *P. acnes*-induced lesions.

### 2.9 Data analysis

The data were entered into a database using Microsoft Excel and statistical analyses were performed using SPSS V.20.0 (IBM, Armonk, NY, USA). To compare categorical data that was presented as frequencies and percentages, the *χ*^2^ test was applied. Statistical significance was determined by two-sided *p* values ≤0.05.

## 3 Results

### 3.1 Baseline characteristics

As shown in Table 1, a total of 94 *P. acnes* isolates were consecutively recovered from patients with AV attending dermatology clinics at the hospital from August 2021 to October 2022. The patients were 12–38 years of age (mean age, 25.6 years). Among the 94 patients, 44 (48.9%) were ≤25 years old, and 50 (51.1%) were >25 years old. The cohort included 58 (61.7%) females and 36 (38.3%) males. According to the acne grading system, 43 (45.7%) patients were graded ≤3, and 51 (54.3%) were graded to be 4. A total of 58 (61.7%) patients were included from 2021, and 36 (38.3%) were from 2022.

**Table 1:** Baseline characteristics of the patients with acne vulgaris.

Characteristics	Patients with acne (*n* = 94)
Age (years)	
≤25	44 (48.9)
>25	50 (51.1)
Gender	
Female	58 (61.7)
Male	36 (38.3)
Disease severity	
≤3 grade	43 (45.7%)
4 grade	51 (54.3%)
Clinics visit time	
2021	58 (61.7)
2022	36 (38.3)

Data were expressed as *n* (%).

### 3.2 Antibiotic susceptibility

**Figure 2:**
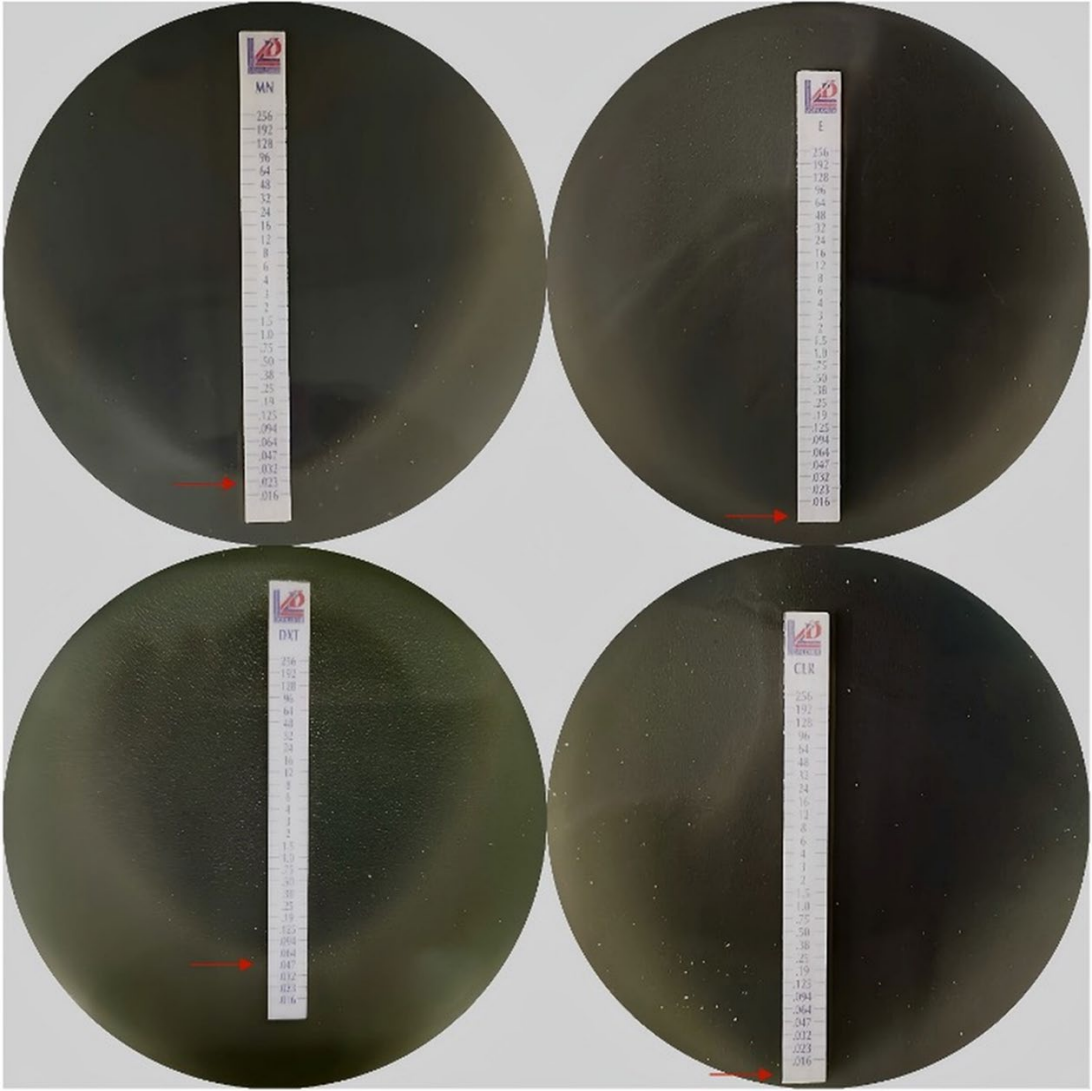
Antibiotics susceptibility using *E*-test method. MN, Minocycline; E, Erythromycin; DMT, Doxycycline; CLR, Clarithromycin; Each panel represents the MIC of an antibiotic as indicated by the intersection of the elliptical zone of inhibition with the *E*-test strip. The red arrows point to the MIC values on each strip.

As shown in Figure 2, the susceptibility of all the *P. acnes* isolates to four antibiotics was assessed using *E*-test method. This figure demonstrates the susceptibility of *P. acnes* isolates to erythromycin, clarithromycin, doxycycline, and minocycline, providing crucial insights for effective treatment strategies.

According to Table 2, all *P. acnes* isolates were found to be susceptible to minocycline and doxycycline. Additionally, 53 (56.4%) and 52 (55.3%) of the isolates were susceptible to erythromycin and clarithromycin, respectively. It was observed that the *P. acnes* collected from patients who were 25 years old or younger exhibited a lower susceptibility to erythromycin (44.4% vs. 67.3%) and clarithromycin (44.4% vs. 65.3%) compared to patients older than 25 years (*p* < 0.01). Furthermore, the *P. acnes* isolates from patients with a grade of 3 or lower were less resistant to erythromycin (20.9% vs. 62.4%) and clarithromycin (20.9% vs. 64.7%) compared to patients with a grade higher than 4 (*p* < 0.01). In terms of gender, 82.8% of all *P. acnes* isolates from females had a minimum inhibitory concentration (MIC) greater than 0.032 mg/L, while only 61.5% of all isolates from males had MIC greater than 0.032 mg/L. This difference was found to be statistically significant (*p* < 0.01). According to Table 3, *P. acnes* isolates with a MIC less than or equal to 0.032 mg/L showed varying levels of susceptibility to four antibiotics: erythromycin, clarithromycin, minocycline, and doxycycline. Specifically, isolates with an MIC lower than 0.032 mg/L exhibited lower susceptibility to clarithromycin compared to isolates with an MIC higher than 0.032 mg/L (49.3% vs. 72.0%, *p* = 0.05).

**Table 2:** Four antibiotics susceptibility of *P. acnes* isolates.

	Erythromycin		Clarithromycin		Minocycline		Doxycycline	
	Susceptible	Resistant	Susceptible	Resistant	a	b	a	b
2021	32 (55.2%)	26 (45.6%)	32 (55.2%)	26 (44.8%)	39 (68.4%)	19 (31.6%)	14 (24.1%)	44 (75.9%)
2022	21 (58.3%)	15 (41.7%)	20 (55.5%)	16 (44.4%)	30 (83.3%)	6 (16.7%)	6 (16.7%)	30 (83.3%)
*χ*^2^ test	*χ*^2^ = 0.090, *p* = 0.764		*χ*^2^ = 0.001, *p* = 0.971		*χ*^2^ = 2.946, *p* = 0.086		*χ*^2^ = 0.740, *p* = 0.390	
Female	34 (58.6%)	24 (41.4%)	34 (58.6%)	24 (41.4%)	42 (72.4%)	16 (27.6%)	10 (17.2%)	48 (82.8%)
Male	19 (52.8%)	17 (47.2%)	18 (50.0%)	18 (50.0%)	27 (77.1%)	9 (22.9%)	10 (38.4%)	16 (61.5%)
*χ*^2^ test	*χ*^2^ = 0.308, *p* = 0.579		*χ*^2^ = 0.668, *p* = 0.414		*χ*^2^ = 0.076, *p* = 0.783		***χ***^**27**^ **= 4.456, *p* = 0.035**	
≤25 y	20 (44.4%)	24 (55.6%)	19 (44.4%)	25 (55.6%)	35 (76.1%)	9 (23.9%)	10 (22.7%)	34 (77.3%)
>25 y	33 (67.3%)	17 (32.7%)	33 (65.3%)	17 (34.7%)	34 (68.0%)	16 (32.0%)	10 (20.0%)	40 (80.0%)
*χ*^2^ test	***χ***^**2**^ **= 4.017, *p* = 0.045**		***χ***^**2**^ **= 4.930, *p* = 0.026**		*χ*^2^ = 1.598, *p* = 0.206		*χ*^2^ = 0.101, *p* = 0.747	
≤Grade 3	34 (79.1%)	9 (20.9%)	34 (79.1%)	9 (20.9%)	30 (69.8%)	13 (30.2%)	9 (20.9%)	34 (79.1%)
Grade 4	19 (37.3%)	32 (62.7%)	18 (35.3%)	33 (64.7%)	39 (76.5%)	12 (23.5%)	11 (21.6%)	40 (78.4%)
*χ*^2^ test	***χ***^**2**^ **= 16.587, *p* = 0.000**		*χ*^2^ **= 18.088, *p* = 0.000**		*χ*^2^ = 0.537, *p* = 0.464		*χ*^2^ = 0.006, *p* = 0.940	
Total	53 (56.4%)	41 (43.6%)	52 (55.3%)	42 (44.7%)	69 (75.8%)	25 (24.2%)	20 (21.3%)	74 (78.7%)

Data were expressed as *n* (%). a: MIC ≤ 0.032 mg/L; b: MIC > 0.032 mg/L. Results of statistical significance are in bold font.

**Table 3:** Interaction of susceptibility to four antibiotics in *P. acnes* isolates.

		Erythromycin		Clarithromycin	
		Susceptible	Resistant	Susceptible	Resistant
Minocycline	a	35 (50.7%)	34 (49.3%)	34 (49.3%)	35 (50.7%)
	b	18 (72.0%)	7 (28.0%)	18 (72.0%)	7 (28.0%)
		*χ*^2^ = 3.378, *p* = 0.066		***χ***^**2**^ **= 3.383, *p* = 0.050**	
Doxycycline	a	12 (60.0%)	8 (40.0%)	10 (50.0%)	10 (50.0%)
	b	41 (55.4%)	33 (44.6%)	42 (56.8%)	32 (43.2%)
		*χ*^2^ = 0.135, *p* = 0.713		*χ*^2^ = 0.291, *p* = 0.590	
	*χ*^2^ test	*χ*^2^ = 0.022, *p* = 0.883			

Data were expressed as *n* (%). a: MIC ≤ 0.032 mg/L; b: MIC > 0.032 mg/L. Results of statistical significance are in bold font.

### 3.3 *Propionibacterium acnes* phage cleavage *in vitro*

**Figure 3:**
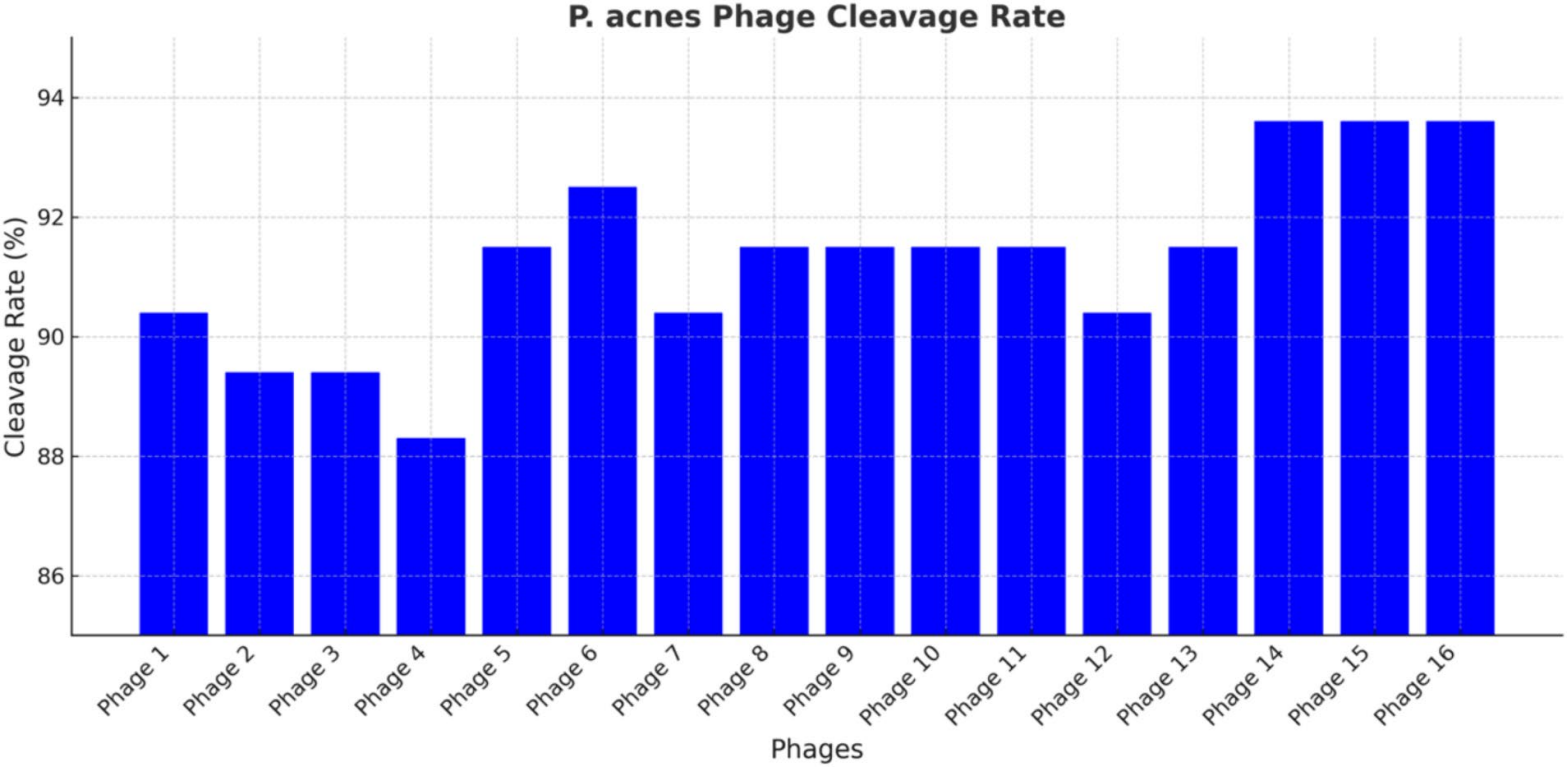
*P. acnes* phage cleavage rate. Each bar represents a different phage, with the *y*-axis indicating the percentage of bacterial lysed. The data highlight the efficiency of each phage in lysing *P. acnes*, identifying the most promising candidates for phage therapy.

This study included a total of 16 *P. acnes* phages, as depicted in Figure 3. The observed cleavage rates ranged from 88.30 to 93.60%, with a mean of 91.6%. Interestingly, the susceptibility rates of *P. acnes* to erythromycin and clarithromycin were considerably lower at 56.4 and 55.3%, respectively, when compared to the cleavage rates of *P. acnes* phages. Conversely, the susceptibility rates of *P. acnes* to minocycline and doxycycline were both 100%, surpassing the cleavage rates of *P. acnes* phages. The pahge15 was selected to apply to treat the acne mouse model. These findings hold significance in both professional and academic settings.

**Figure 4:**
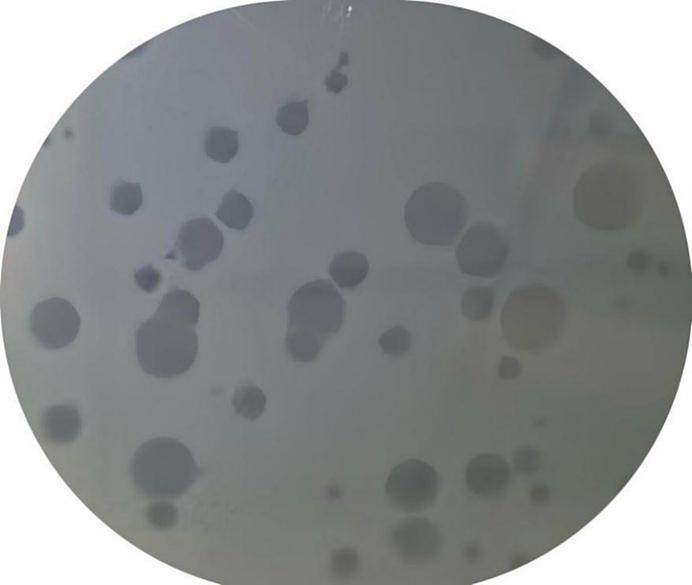
*P. acnes* phage plaque. The clear zones, known as plaques, indicate areas where phages have lysed the bacterial.

As shown in Figure 4, phages demonstrated the ability to cleavage the *P. acnes*. The clear zones, known as plaques, indicate areas where phages have lysed the bacteria. These plaques are crucial for evaluating the phage’s ability to infect and lyse *P. acnes*. The size, number, and clarity of the plaques provide important information about the phage’s lytic activity and potential efficacy in phage therapy against *P. acnes* infections.

### 3.4 MLST analysis of *Propionibacterium acnes* isolates

MLST analysis was performed on eight randomly selected *P. acnes* isolates from the study. The analysis revealed diverse genetic profiles among the isolates, with specific allelic patterns identified for each of the eight housekeeping genes (aroE, atpD, gmk, guaA, lepA, sodA, tly, and CAMP2). Table 4 provides a detailed summary of the allelic profiles, sequence types (ST), clonal complexes, and lineage types for each isolate. The MLST analysis identified four of the isolates as belonging to the IA-2 subtype. Among these, the NO1 isolate with the IA-2 subtype was selected for further experimentation in addressing *P. acnes*-induced lesions in mice.

**Table 4:** MLST analysis of eight *P. acnes* isolates.

NO	aroE	atpD	gmk	guaA	lepA	sodA	tly	CAMP2	MLST	Clonal complex	Lineage type
1	1	1	1	3	1	1	2	3	ST-31	CC3 (type IA1)	I-A1
2	1	1	1	5	1	4	8	2	ST-2	CC2 (type IA2)	I-A2
3	1	1	1	3	1	1	22	2	ST-115	CC3 (type IA1)	I-A1
4	17	4	2	4	4	6	10	46	ST-153	/	/
5	1	1	1	5	1	4	8	2	ST-2	CC2 (type IA2)	I-A2
6	1	1	1	3	1	1	22	2	ST-115	CC3 (type IA1)	I-A1
7	1	1	1	5	1	4	8	2	ST-2	CC2 (type IA2)	I-A2
8	1	1	1	5	1	4	8	2	ST-2	CC2 (type IA2)	I-A2

NO, The number assigned to each isolate; aroE, atpD, gmk, guaA, lepA, sodA, tly, CAMP2, The alleles for each housekeeping gene used in MLST analysis; Clonal complex, A group of related sequence types; Lineage type, The broader lineage classification based on the MLST data.

### 3.5 Histopathological evaluation of *Propionibacterium acnes*-induced lesions in mice

**Figure 5:**
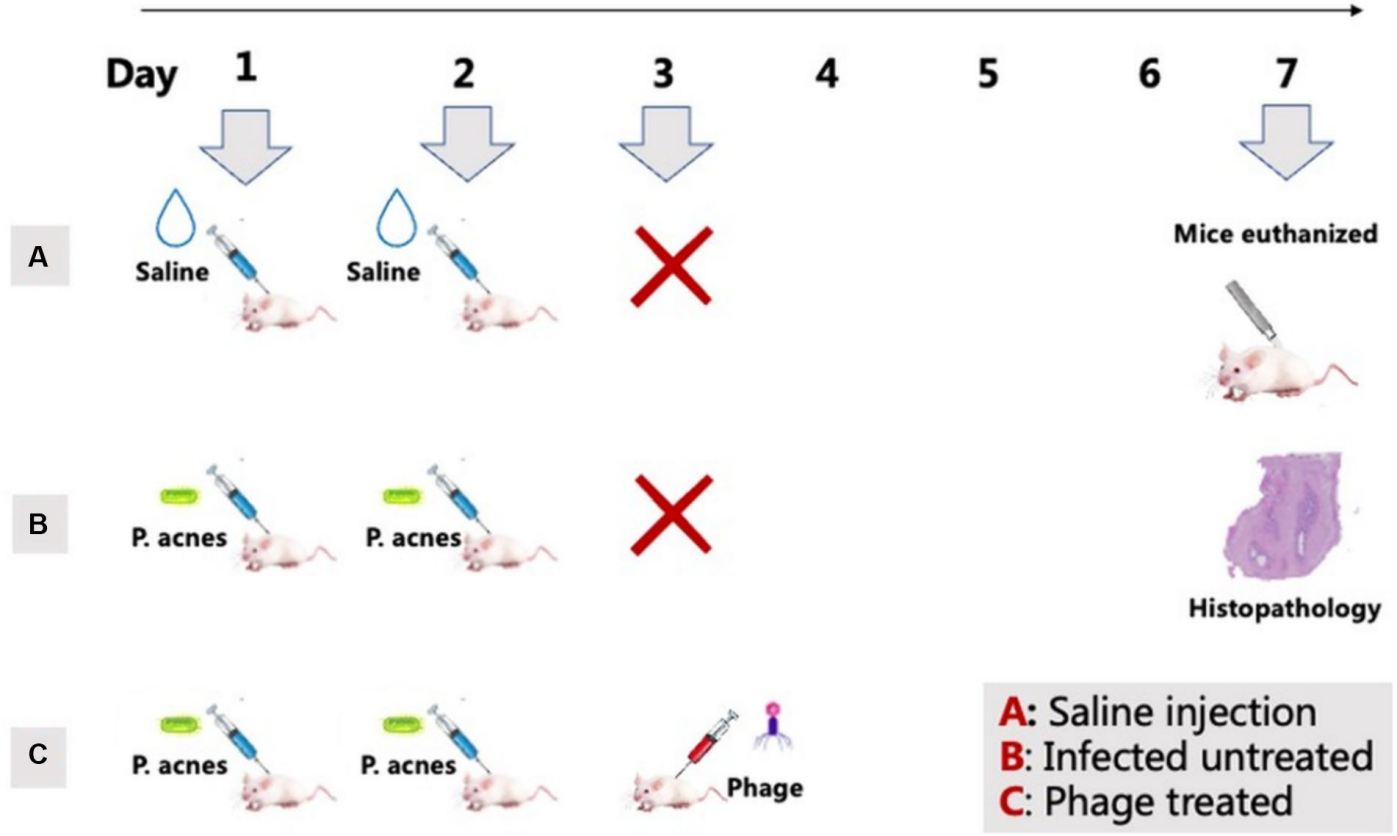
*P. acnes*-induced lesions procedure. Group A: Saline injections on days 1 and 2 (control). Group B: *P. acnes* injections on days 1 and 2 (infected untreated). Group C: *P. acnes* injections on days 1 and 2, followed by phage treatment on day 3 (phage treated).

**Figure 6:**
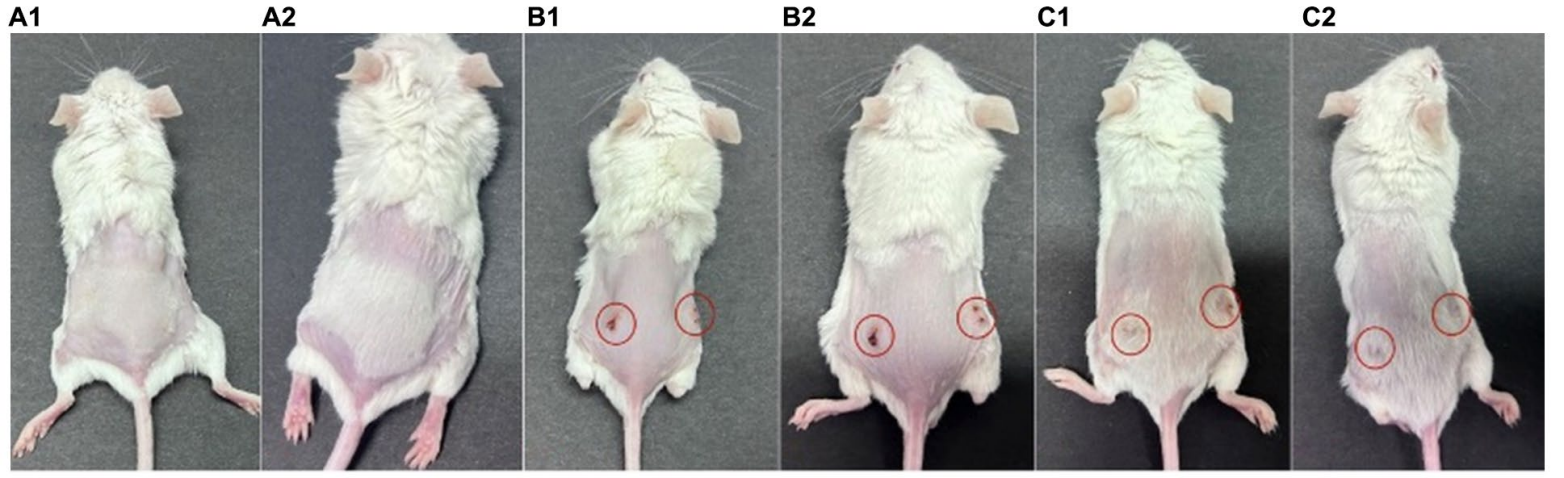
*P. acnes*-induced lesions in mice. **(A)** Control group with saline injection. **(B)** Infected untreated group, showing lesions marked with red circles. **(C)** Phage-treated group, with lesions marked and showing potential improvement.

The procedure of *P. acnes*-induced lesions is shown in Figure 5. In group A, there were no observed inflammatory papules (Figures 6A1–B2). Group B exhibited noticeable inflammatory papules (Figures 6B1,B2), and group C also presented prominent inflammatory papules (Figure 6C1). Following phage 15 therapy, the inflammatory papules in Figure 6C2 was less significant compared to Figure 6C1.

**Figure 7:**
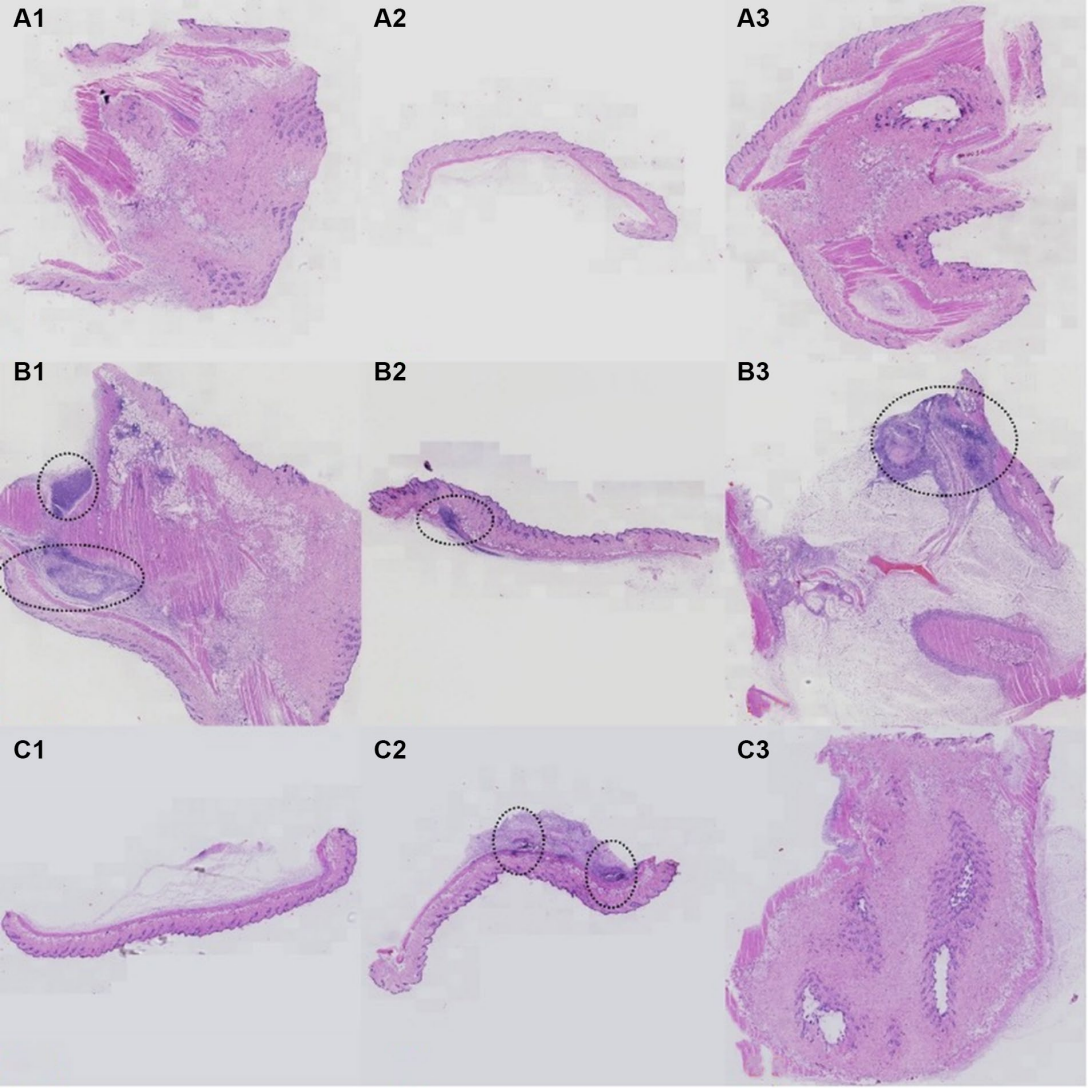
Histopathology results of *P. acnes*-induced lesions in mice. **(A)** Control group with normal skin histology. **(B)** Infected untreated group showing significant inflammation and tissue damage (highlighted by arrows). **(C)** Phage-treated group exhibiting reduced inflammation and tissue damage compared to the untreated group.

Detailed information of histopathological results were showed in Figure 6. In group A, mice received saline injections, and histopathological analysis revealed no signs of inflammatory cell infiltration in Figures 7A1–A3. For group B, mice injected with *P. acnes* without phage therapy exhibited distinct clustering infiltration of inflammatory cells within the circled area in Figures 7B1–B3. In group C, mice injected with *P. acnes* and subjected to phage therapy after 3 days displayed clustering infiltration of inflammatory cells in Figure 7C2, while Figures 7C1,C3 did not show similar infiltration. Notably, Figures 7B1–B3 exhibited a more pronounced infiltration.

## 4 Discussion

*Propionibacterium acnes* primarily resides in the pilosebaceous unit of the skin. It stimulates the production of IL-1, influences the growth and differentiation of keratinocytes, and contributes to the formation of comedones, which are a hallmark of AV (Isard et al., 2011). Through immunological reactions, *P. acnes* also triggers the release of proinflammatory substances by sebocytes, keratinocytes, and peripheral blood mononuclear cells (Thiboutot et al., 2014). The role of interleukins (ILs) and toll-like receptors (TLRs) in modulating immune responses and inflammation is significant in the context of AV. IL-1, as part of the IL-1 superfamily cytokines, has been shown to play critical roles in immune responses and inflammatory conditions (Behzadi et al., 2022). TLRs, particularly TLR2, recognize *P. acnes* and initiate inflammatory signaling pathways, leading to the production of cytokines and chemokines that contribute to AV pathogenesis (Mukherjee et al., 2023). These immune responses can exacerbate the inflammatory environment of AV lesions. Additionally, *P. acnes* is associated with increased production of lipids, exacerbating inflammation in AV (Del Rosso et al., 2008). These findings suggest that *P. acnes* may play a crucial role in the development of AV. For over 50 years, antibiotics targeting *P. acnes* have been the mainstay of AV treatment, believed to primarily work by inhibiting *P. acnes* growth and reducing inflammation.

According to current guidelines, the most recommended oral treatments for AV include isotretinoin and antibiotics such as erythromycin, clarithromycin, minocycline, and doxycycline (Zaenglein et al., 2016). However, it is important to note that isotretinoin should not be combined with tetracyclines due to the risk of lip exfoliative dermatitis and hyperglyceridemia. In addition, the use of isotretinoin can lead to premature epiphyseal closure and bone demineralization (Luthi et al., 2012). It is worth mentioning that dermatologists prescribe approximately 60% of all antibiotic prescriptions to AV patients (Del Rosso et al., 2008). For mild and moderate AV, topical antibiotics are often used as a first-line treatment, although there is a growing trend toward the use of oral antibiotics. Surprisingly, a significant number of AV patients (64%) have previously undergone antibiotic therapy, sometimes even with the simultaneous administration of two different antibiotics (Zhu et al., 2019). In China, oral and topical antibiotics are commonly used for the treatment of AV, based on our experiences. Furthermore, research has shown that individuals who received antibiotic treatment (both topical and oral) had higher rates of antibiotic-resistant *P. acnes* and higher MICs compared to those who did not receive antibiotic treatment (Zhu et al., 2019).

Therefore, dermatologists ought to utilize caution while prescribing antibiotics because appropriate and reasonable antibiotic use would reduce antibiotic resistance. Antibiotic overuse and misuse have a significant influence on the development of antibiotic resistance (Centers for Disease Control and Prevention, 2013). This occurrence may be attributed to inducible resistance as well as the widespread administration of macrolide antibiotics, particularly for respiratory infections. Tetracycline and Doxycycline, on the other hand, are used majorly for AV, other skin infections, and sexually transmitted diseases (STDs), with a more limited spectrum of use, resulting in a lower resistance rate (Zhu et al., 2019). Currently, individuals suffering from AV generally receive lengthy courses (6–8 weeks) of a single antibiotic, which exposes them to the drug at various concentrations and has the potential to result in resistance (Lee et al., 2014). It has been shown that combining antibiotics with retinoids or benzoyl peroxide (BPO) treatment decreases antibiotic resistance compared with antibiotics alone (Nast et al., 2012). As a result, the Global Alliance to Improve Acne Outcomes Group suggested that antibiotics be used for short periods, together with retinoid and BPO but no other antibiotics, and not used as monotherapy or as maintenance therapy.

Drug resistance in *P. acnes* has gradually become a major concern due to the increased consumption of various antibiotics. Multiple studies on *P. acnes*’ resistance to antibiotics have been published in several countries. One study estimated the prevalence of antibiotic resistance in Spain to reach 94% (Ross et al., 2003). In addition, a UK study found a definite rise in the proportion of *P. acnes* strains resistant to antibiotics, from 34.5% in 1991 to 64% in 1997 (Coates et al., 2002). A recent Japanese study revealed that the severity of acne is correlated with an increase in *P. acnes* antibiotic resistance (Nakase et al., 2014). Individuals in Korea who have previously received oral or topical antibiotic treatment have higher MICs to doxycycline than individuals who have not received antibiotic treatment (Song et al., 2011). Data from earlier investigations on *P. acnes* resistance conducted in China revealed that lincomycin and macrolides are facing a critical condition of resistance (Fan et al., 2016). As demonstrated in this investigation, *P. acnes* isolates were highly susceptible to tetracyclines (doxycycline and tetracycline), but highly resistant to erythromycin and clarithromycin. In the sub-group analysis, AV patients under the age of 25 had higher levels of erythromycin and clarithromycin resistance than those above the age of 25. The Grade 4 AV sufferers also experienced this circumstance. Compared to male patients, female patients were less susceptible to doxycycline.

Therefore, it is crucial to find effective alternatives to widely used antibiotics to reduce the risk of resistance and obtain a specialized treatment that can effectively eliminate *P. acnes*. Phages, also known as “living drugs,” have been recognized by the NIH as a novel approach to combat antibiotic resistance (Jassim and Limoges, 2014). Data supports their effectiveness in treating both local and systemic infections caused by antibiotic-resistant bacterial strains (Schrom et al., 2016). Targeted therapies for AV, such as bacteriophages, can provide significant economic savings alongside clinical benefits. By specifically targeting *P. acnes*, these therapies reduce the risk of antibiotic resistance, which is a major cost factor in healthcare. Although the initial costs of developing and administering targeted therapies might be higher, they lead to substantial long-term savings. This includes reducing the need for prolonged antibiotic treatments, decreasing hospitalizations due to severe acne or complications, and minimizing the overall burden on healthcare systems. In this study, the *P. acnes* phages demonstrated high cleavage rates, ranging from 88.30 to 93.60% (mean, 91.6%), indicating their sensitivity to *P. acnes in vitro*. Phages have several advantages over antibiotics, including their specificity to their bacterial host which reduces the risk of secondary infections, their ability to multiply at the site of infection where vulnerable bacteria are present, their natural occurrence through natural selection making them environmentally friendly, and their ability to be isolated for therapeutic use (Golkar et al., 2014).

*P. acnes* genotypic subtypes IA-2, IB-1, and IC are linked to AV. Specifically, IA-2 subtype, IB-3, II, and III subtypes are associated with infections in prosthetic joints, spine, and other tissues (Johnson et al., 2016). In the study by McDowell et al., it was observed that phylotype IA-2 strains, identified by MLST CC3, and a smaller number of IA-1 strains (CC1) constituted the majority of strains resistant to tetracycline, erythromycin, and clindamycin. Additionally, all tested IC strains exhibited resistance to at least one of these antibiotics (McDowell et al., 2012). Similarly, Lomholt and Kilian reported elevated resistance rates in phylotype IA-2, IA-1, and potentially IB-1 strains (Lomholt and Kilian, 2014).

In this study, our aim was to assess the potential of bacteriophage therapy in a *P. acnes*-induced mice model. To achieve this, we induced *P. acnes*-derived skin lesions successfully by administering consecutive injections of *P. acnes* NO1. The absence of lesions when injecting physiological saline instead of bacteria further validated the model. The skin lesions did not exhibit exact clinical and histopathological characteristics of human AV. While comedones are typically observed in common acne, our model displayed deep subcutaneous infiltration in the lesions, possibly due to intradermal injection of *P. acnes*, resembling inflammatory nodules seen in severe AV (Rimon et al., 2023). However, the purpose of establishing this model was to evaluate bacteriophage therapy, which can reduce bacterial levels and subsequently mitigate the severity of lesions. The treated group showed significant improvements lymphocyte mass in compared to the untreated group. Nevertheless, further confirmation of these results requires additional experimentation with more mice.

The study has several limitations. First, the sample size was small and all participants were from a single center, which may limit the generalizability of the findings. Second, the cross-sectional design of the study makes it difficult to establish causality. It would be beneficial to conduct multicenter trials to gain a more comprehensive understanding of *P. acnes* antibiotic resistance. Third, there is a lack of data on the impact of macrolides and tetracycline medications on bacterial rRNA elements. Future research should focus on amplifying and sequencing bacterial resistance-related genes. Four, the study did not utilize animal models to assess the effectiveness of the *P. acnes* phage. This is an area that should be explored in future investigations. Fifth, the number of animals in this experiment is small, and it is only a small-scale experiment. It is necessary to increase the number of animals and analyze the test results through statistical analysis to confirm the effectiveness of phage on *P. acnes*. Sixth, the inclusion criteria did not record whether the participants had used topical or systemic antibiotic therapy in the previous 3 months. This factor should be considered and documented in future studies. Lastly, phage resistance is another limitation that must be considered and warrants further study.

Our study presents several notable strengths. Firstly, we employed a comprehensive approach, analyzing both the antibiotic susceptibility of *P. acnes* and the effectiveness of phage therapy through detailed *in vitro* and *in vivo* experiments. This robust methodology ensures the reliability of our findings. Secondly, the diverse collection of *P. acnes* strains from patients of varying demographics and acne severities provides a representative overview of resistance patterns in the Beijing region. This data is crucial for understanding local antibiotic resistance trends and guiding effective treatment strategies. Moreover, our exploration of bacteriophage therapy highlights a promising alternative to traditional antibiotics, especially given the rising issue of antibiotic resistance. The high phage cleavage rates observed underscore the potential of phage therapy in treating *P. acnes*-induced lesions. Lastly, our study’s findings have significant clinical implications. By documenting detailed susceptibility profiles and demonstrating the efficacy of phage therapy, we provide valuable insights that could enhance therapeutic approaches for acne vulgaris and help combat antibiotic resistance.

## 5 Conclusion

Doxycycline and tetracycline showed strong effectiveness against *P. acnes*, while erythromycin and clarithromycin had high resistance rates. This indicates that each doctor including dermatologists should exercise caution when prescribing antibiotics for AV patients. It is important to explore alternative therapies to antibiotics, such as *P. acnes* phages, which demonstrated high susceptibility to *P. acnes* isolates. Further research should be conducted to investigate these potential alternatives.

## Data availability statement

The raw data supporting the conclusions of this article will be made available by the authors, without undue reservation.

## Ethics statement

The studies involving humans were approved by China-Japan Friendship Hospital. The studies were conducted in accordance with the local legislation and institutional requirements. The participants provided their written informed consent to participate in this study. The animal study was approved by China-Japan Friendship Hospital. The study was conducted in accordance with the local legislation and institutional requirements.

## Author contributions

RXY: Writing – original draft, Writing – review & editing. LYY: Data curation, Methodology, Project administration, Writing – review & editing. XLN: Data curation, Methodology, Software, Writing – review & editing. YC: Conceptualization, Investigation, Writing – review & editing.
